# Food (Matrix) Effects on Bioaccessibility and Intestinal Permeability of Major Olive Antioxidants

**DOI:** 10.3390/foods9121831

**Published:** 2020-12-09

**Authors:** Dubravka Vitali Čepo, Kristina Radić, Petra Turčić, Dora Anić, Barbara Komar, Mirela Šalov

**Affiliations:** Faculty of Pharmacy and Biochemistry, University of Zagreb, Ante Kovačića 1, 10000 Zagreb, Croatia; dvitali@pharma.hr (D.V.Č.); pturcic@pharma.hr (P.T.); danic@student.pharma.hr (D.A.); bkomar@student.pharma.hr (B.K.); msalov@pharma.hr (M.Š.)

**Keywords:** olive pomace, hydroxytyrosol, tyrosol, polyphenol-food interaction, Caco-2 monolayer, in vitro digestion

## Abstract

Background: olive pomace extract (OPE) is a rich source of health promoting polyphenols (hydroxytyrosol (HTS) and tyrosol (TS)) and can be used as a nutraceutical ingredient of dietary supplements and functional foods. Its adequate bioavailability is a prerequisite for excreting biological activity and can be significantly and specifically affected by different food matrices. Methods: in order to investigate food effects on polyphenol bioaccessibility, OPE was co-digested with different foods according to internationally harmonized in vitro digestibility method. Impact of particular nutrients on HTS and TS permeability was assessed on Caco-2 cell monolayer. Results: HTS and TS bioaccessibility and transepithelial permeability can be significantly affected by foods (nutrients), especially by casein and certain types of dietary fiber. Those effects are polyphenol-and nutrient-specific and are achieved either through complexation in gastrointestinal lumen and/or through direct effects of nutrients on intestinal monolayer. Conclusions: obtained results emphasize the significance and complexity of polyphenol interactions within the food matrix and the necessity of individual investigational approaches with respect to particular food/nutrient and interacting phenolic compounds.

## 1. Introduction

The well-established health effects of olive oil can be attributed to its high monounsaturated fatty acid content; it is principally made of oleic acid (56% to 84% of the total fraction of fatty acids) and its phenolic compounds are effective at decreasing the risk of cardiovascular disease, mostly due to its ability to reduce the peroxidation of blood lipids [1,2]. The main antioxidants in olive oil are two groups of phenolic compounds: lipophilic and hydrophilic. While lipophilic phenols, including tocopherols and tocotrienols, can be found in other types of vegetable oils, hydrophilic phenols (hydroxytyrosol, tyrosol, and oleuropein) are characteristic of olive oil, and are not found in other oils and fats. They demonstrate unusual sensory properties, exert antioxidant, immunomodulatory, antimicrobial, and anticancer activity, and may decrease the risk of numerous, chronic, non-infectious diseases, such as atherosclerosis, different types of cancer (colorectal, prostate, breast, etc.), chronic inflammation, strokes (e.g., ischemic stroke), and other degenerative diseases (primarily neurodegenerative diseases, such as Alzheimer’s disease) [2]. Olive pomace and olive pomace wastewater, the residues left after the production of olive oil, are secondary raw materials that contain significantly higher amounts of olive polyphenols compared to olive oil, particularly hydroxytyrosol (HTS) and tyrosol (TS). Numerous green extraction methods for efficient extraction of polyphenols from olive mill waste were recently developed [3,4,5,6], and significant steps forward have been taken in developing functional formulations of olive pomace extracts (OPEs) that will ensure satisfactory composition, stability, bioavailability, and biological activity of major active compounds, primarily HTS and TS [7,8].

Bioavailability of polyphenols is essential for their biological activity. Since it differs greatly among various phenolic compounds and depends on the characteristics of the food source, polyphenols that are the most abundant in food are not necessarily the ones with the most important health-related impacts. Since the current knowledge on health-effects of polyphenols arise mainly from epidemiological studies, it is necessary to gain better insight into their bioavailability in order to establish conclusive evidence for their effectiveness in disease prevention [9].

Bioavailability can be defined as the fraction of aa particular compound available for physiological functions in the body. In the case of polyphenols, it depends on release of polyphenols from the food matrix and/or interactions with meal (1); structural changes that occur during gastro-intestinal digestion (2); cellular uptake of glycoside forms of polyphenols and respective aglycons by enterocytes (3); fermentation of nonabsorbed polyphenols by gut microbiota and formation and absorption of metabolites (4); and metabolism of all absorbed compounds (5) [10].

Impact of food matrix (food components) on bioavailability of nutraceutical is particularly important in cases when it is necessary to compare different food matrices as the sources of the same bioactive compounds (for example olive oil vs. OPE as sources of HTS and TS). Assessing polyphenol-food interactions is also of great importance because nutraceuticals may be isolated from their natural environment, purified, and then used as nutraceutical ingredients in processed (functional) food [11]. Additionally, sufficient knowledge on the significance and the type of polyphenol-food (nutrient) interactions enables targeted use of absorption, promoting interactions and subsequent formulation of excipient foods that would improve bioavailability of particular polyphenols [12].

Mechanisms of polyphenol–food interactions are numerous. Presence of food in the gastrointestinal system changes the physiological conditions in the human gastrointestinal tract (fluid volumes, gastric, and intestinal motility, gastric emptying, luminal pH values, enzyme capacity, osmolality, bile salt content) and, consequently, affects stability and solubility of particular phenolic compounds. Moreover, polyphenols can form complexes with nutrients (components of the meal) such as dietary fiber, carbohydrates, lipids, and proteins, which can significantly affect their bioaccessibility. Additionally, nutrients can also interact with the process of absorption of polyphenols through interactions with influx and efflux intestinal transporters, interactions with intestinal monolayer permeability, or interactions with intestinal metabolizing enzymes bioaccessibility [13,14,15].

Bioavailability of HTS and TS from olive oil has been investigated in more in vivo studies and it was found to be satisfactory, with plasma HTS and TS concentrations rising early after virgin olive oil ingestion and reaching a peak at around 1 h in plasma and 0–2 h in urine [16]. It is important to emphasize that complex secoiridoids from olive oil are biotransformed by gut microflora, giving additional rise to postprandial HTS and TS [17,18]. OPEs are generally richer sources of HTS and TS in comparison to extra-virgin olive oil, but they have rarely been investigated in terms of bioavailability. Recent data obtained by Radić and co-authors [8] show that OPE polyphenols are stable during gastrointestinal digestion (88–187%; due to degradation of secoiridoids and formation of HTS and TS during digestion). Investigation of permeability showed that HTS and TS are primarily absorbed by passive diffusion and that absorbed oleuropein is extensively metabolized in Caco-2 cells. OPE matrix negatively influenced the permeability of HTS and TS, but the negative effect could be partially neutralized by absorption-promotive effects of particular cyclodextrins [8].

This research builds up on the work of Radić and co-authors [8], and its main focus is to investigate the impact of particular foods on gastrointestinal bioaccessibility and permeability of HTS and TS from OPE. As mentioned before, functional extracts, rich in bioactive health promoting compounds, such as OPE, are nowadays being extensively used as nutraceutical ingredients of dietary supplements and functional foods. It is therefore of great importance to understand the nature and significance of polyphenol–food interactions in order to provide efficient and accurate dosing instructions and enable targeted formulation of dietary supplements or excipient foods that will result in optimized bioavailability of those health-promoting compounds.

## 2. Materials and Methods

### 2.1. Chemical and Reagents

Petrol ether, dimethyl sulfoxide (DMSO) ethanol, methanol (≥99.9%), sodium acetate, Folin-Ciocalteu reagent, Dulbecco’s Phosphate Buffered Saline (PBS liquid, sterile-filtered, without calcium, without magnesium, suitable for cell culture), tert-butyl hydroperoxide (tBOOH), 3-hydroxytyrosol (HTS) and tyrosol (TS) (≥98%), bile salts, thermostable α-amylase (A3306), pancreatin from porcine pancreas (4 × USP), Dulbecco’s Modified Eagle’s Medium, D-glucose, ethylenediaminetetraacetic acid (EDTA), and sodium caseinate were from Sigma–Aldrich (St. Louis, MO, USA). Acetonitrile (≥99.9%) was from Honeywell (Charlotte, NC, USA). Acetic acid and Na_2_CO_3_ were from Kemika (Zagreb, Croatia). Pepsin (from porcine gastric mucosa) 0.7 FIP-U/mg was from Merck (Darmstadt, Germany). Heat inactivated fetal bovine serum (FBS), nonessential amino acids (NEAA), penicillin/streptomycin/amphotericin B (A/A), and trypsin were from Capricorn Scientific (Ebsdorfergrund, Germany). The 3-(4,5-dimethylthiazole-2-yl)-2,5-diphenyltetrazolium bromide (MTT) was from Panreac AppliChem (Darmstadt, Germany). Caco-2 cells were from American Type Culture Collection (ATCC, Manassas, VA, SAD). Hydroxypropyl β cyclodextrin (HPB) was purchased from Wacker–Chemie GmbH (Burghausen, Germany), Cellulose Alba Fiber^®^C200 from Mikro-Technik (Bürgstadt, Germany), and Inulin Orafti^®^HSI from Beneo (Mannheim, Germany). Pectin was from Foodchem (Zhangjiang, China). Ultrapure water (18 MΩ) was obtained from SG Reinstwassersystem Ultra Clear UV Plus coupled with SG Wasservollentsalzer-Patrone SG 2800 (Günzburg, Germany). Sunflower oil and full-fat milk and food matrices were obtained from local suppliers, unless noted otherwise. All other chemicals were from Kemika (Zagreb, Croatia).

The exact composition of simulated salivary fluid (SSF), simulated gastric fluid (SGF), and simulated intestinal fluid (SIF) is presented in Table S1. Fed state simulated gastric fluid (FeSSGF) contained sodium chloride (237 mM), acetic acid (17.1 mM), and sodium acetate (29.8 mM) and was mixed with full fat milk in 1:1 ratio. The pH was adjusted to 3 by using hydrochloric acid. Fed state simulated intestinal fluid (FeSSIF) contained CaCl_2_ (1.67 mM), MgSO_4_ (0.81 mM), KCl (5.37 mM), KH_2_PO_4_ (0.44 mM), NaHCO_3_ (0.42 mM), NaCl (137 mM), Na_2_HPO_4_ (0.34 mM), D-glucose (5.55 mM), L-glutamine (2 mM), lecithin (7.5 mM), sodium taurocholate (1 mM) and MES (1 M) to adjust pH to 6. Standardized food model (SFM) was prepared according to the procedure of Zhang and co-workers [19] with slight modifications (Table S2). Briefly, sodium caseinate (1%, *w/w*) was dissolved in phosphate buffer solution (10 mM, pH 7) and then filtered to remove any residual insoluble matter. Sunflower oil was gradually added (7.6%, *w/w*) and vortexed for 5 min. Powdered sodium caseinate was gradually added into the emulsion to reach a final concentration of 7.7% *w/w* protein and continuously stirred for 30 min. An aqueous pectin (1.6% *w/v*) solution was obtained by dispersing pectin powder into distilled water and then mixing at 50 °C for 60 min. The mixture was then cooled to room temperature and stirred continuously to fully dissolve the pectin. Then 44.8 g of the prepared pectin solution was mixed with 44.8 g of emulsion (1:1, *w/w*). At the end, 5.15 g of corn starch was slowly poured into the previous mixture with continuous stirring until it was fully dissolved (around 30 min). Pepsin solution was prepared by dissolving 50 mg of pepsin in 1 ml of SGF (25 000 U/mL). Pancreatin solution was prepared by dissolving 8 mg of pancreatin in 1 mL of SIF (800 U/mL). Bile salt solution was prepared by dispersing 65.37 mg of bile salt in 1 mL of water. The α-amylase solution was prepared by dissolving thermostable α-amylase in SSF (1500 U/mL). Hank’s balanced salt solution (HBSS) pH 6.0 was prepared by dissolving KCl (0.4 mg/mL), NaHCO_3_ (0.35 mg/mL), NaCl (8.0 mg/mL), D-glucose monohydrate (1.1 mg/mL), KH_2_PO_4_ (0.06 mg/mL), Na_2_HPO_4_ × 2H_2_O (0.06 mg/mL), CaCl_2_ × 2H_2_O (0.185 mg/mL), MgCl_2_ × 6H_2_O (0.1 mg/mL), MgSO_4_ × 7H_2_O (0.1 mg/mL), and HEPES (7.15 mg/mL) in ultrapure water. All of the solvents needed for chromatographic separation were degassed with Branson 1210 Ultrasonic Cleaner (Danbury, CT, USA) before analysis. Acetate buffer was prepared by mixing sodium acetate 0.1 M:acetic acid 0.1 M (2:1 *v/v*) and adjusting the pH to 5 with pH meter (702 SM Titrino, Metrohm, Herisau, Switzerland).

### 2.2. Preparation of Samples

Olive pomace extract (OPE) was prepared according to previously published procedure [8] with some modifications. Briefly, olive pomace was dried at 60 °C for 24 h in an incubator (INKO, Zagreb, Croatia), sieved through Φ 0.8 mm sieve (Prüfsieb DIN 4188, Kassel, Germany), and defatted with petrol ether using the Soxhlet apparatus (INKO SK6ESS, Zagreb, Croatia). Pre-treated olive pomace was mixed with 20% ethanol (20 g/L) and the extraction was performed by 2 h maceration in a shaking water bath at 70 °C and 100 rpm (Thermostat, Inko, Zagreb, Croatia). The mixture was filtered to remove the crude parts, ethanol was removed from the filtrate under reduced pressure (Rotavapor R-220 EX, BÜCHI Labortechnik AG, Flawil, Switzerland) and obtained water extracts were dried for 48 h in a lyophilizator (Alpha 1-4 LOC-1, Martin Christ Gefriertrocknungsanlagen GmbH, Osterode am Harz, Germany). Dry OPE was used for the simulation of gastrointestinal digestion. Chemical composition of OPE is presented in Table S3. Olive oil (OO) was obtained from the local supplier. Prior to simulation of gastrointestinal digestion OO was diluted with water in 1:10 ratio and vortexed thoroughly (VTY-3000L, Mixer UZUSIO, Tokyo, Japan). For the investigation of polyphenol-food interactions the following materials were used: dietary fiber (cellulose, pectin, inulin), tuna (canned), sour cream (12% milk fat), milk (fresh, 3.2% milk fat), yogurt (2.8% milk fat), milk formula, banana (raw minced), breakfast cereals, soy flakes, fresh cheese, meat sauce, apple (grated, skinless), silver beat (blanched, minced), bread, potato (boiled), honey, and corn starch. Basic nutritive composition was obtained from producers’ nutrition data labels or from nutrition data tables [20] and are presented in supplementary materials (Table S4).

### 2.3. Determination of Total Phenols

Total polyphenols (total reductive capacity) of analyzed samples was assessed by Folin–Ciocalteu method that relies on the transfer of electrons in alkaline medium from phenolic (or other reductive) compounds to phosphomolybdic/phosphotungstic acid complexes, which are determined spectroscopically at 765 nm. Experiments were conducted according to the protocol described by Ainsworth and co-workers [21]. Moreover, 20 µL of adequately diluted sample/standard/solvent (blank) were added in triplicate to 96-well plate (Thermo Fisher Scientific 130188, Rochester, NY, USA) and incubated at 37 °C with 50 µL of Folin Ciocalteu reagent (10% (*v/v*)) for 5 min. The 160 µL of Na_2_CO_3_ (700 mM) was added into each well, shaken, and incubated at 37 °C for 30 min. Absorbance was read at 765 nm and results were expressed as gallic acid equivalents (GAE).

### 2.4. HPLC-FLD Determination of Hydroxytyrosol and Tyrosol

HTS and TS were identified and quantified by HPLC system (Waters Alliance 2695, Milford, MA, USA) coupled with a 2475 Multi λ detector (FLD) with Xenon lamp, according to slightly modified method of Tsarbopoulos and co-workers [22]. Samples were prepared by filtration through 0.45 μm polyethersulfone (PES) syringe filters (Macherey–Nagel, Düren, Germany). Chromatographic separation was conducted by injecting 20 μL of sample on a reversed phase column (250 × 4.6 mm, 5 μm) (Agilent Zorbax Eclipse Plus C18, Santa Clara, CA, USA). Mobile phases were 0.05 M sodium acetate buffer pH 5 and acetonitrile with the flow rate of 1 mL/min. Elution was conducted over 25 min at 25 °C. Identification was performed with FLD set at the excitation wavelength of 280 nm, and emission wavelength of 316 nm. Polyphenols were identified by comparing the retention times of the eluting peaks with those of the standards. Peaks were quantified by using the Empower2 software (Waters, Milford, MA, USA) and compared to external standard calibration. Standard stock solutions were prepared by dissolving reference compounds in DMSO.

### 2.5. Simulation of Gastrointestinal Digestion

In vitro simulation of gastrointestinal digestion and the assessment of bioaccessibility of hydroxytyrosol and tyrosol from OPE was conducted by standardized static in vitro digestion method suitable for food [23]. Three-phase digestion process was applied consisting of short salivary phase, simulation of gastric digestion and simulation of duodenal digestion. Briefly, 200 mg of OPE was used alone or mixed with 600 mg of food matrix (Table S4) in 50 mL Falcon tube. The 875 µL of SSF, 125 µL of salivary α amylase solution, 6.25 µL of CaCl_2_ (0.3 mol/L), and 243.8 µL of water was added, and the mixture incubated at 37 °C for 2 min with constant shaking. The 2.5 mL of reaction mixture were transferred to another Falcon tube and mixed with 1.875 mL of SGF, 1.25 µL of CaCl_2_ (0.3 mol/L), and 50 µL of HCl (1 mol/L). The pH of the mixture was adjusted to 3, and total volume was adjusted to 5 mL with water. The content of the Falcon tube was vortexed and incubated for 2 h at 37 °C with constant shaking. Every 15 min pH of the mixture was checked and adjusted to 3 if necessary. At the end of simulation of the gastric digestion step, 5 mL of aliquot were transferred to another 50 mL Falcon tube for the simulation of intestinal digestion. The 2.75 mL of SIF, 1.25 mL of pancreatin solution, 0.625 mL of bile salt (160 mmol/L), 10 µL of CaCl_2_ (0.3 mol/L), and 37.5 µL of NaOH (1 mol/L) were added. The pH was checked and adjusted to 7. Total volume of reaction mixture was adjusted to 10 with water, mixtures were vortexed and incubated for 2 h at 37 °C with constant shaking. Every 15 min, pH of the mixture was checked and adjusted to 7 if necessary. After digestion, simulation samples were cooled at −20 °C for 10 min and centrifuged (Heraeus Biofuge Stratos, Hanau, Germany) for 20 min at 4 °C and 4100 rpm. For HPLC analysis of hydroxytyrosol and tyrosol, obtained supernatants were additionally filtered through 0.45 µm polyether sulfone (PES) syringe filters. Supernatants were collected and used for the assessment of bioaccessible HTS and TS fractions. Aliquots of samples to be used on cell monolayers were kept at 100 °C for 5 min in Thermomixer R (Eppendorf, Hamburg, Germany) for enzyme inactivation. For each sample blank was prepared, by omitting the addition of OPE to reaction mixture. Simulations of gastrointestinal digestion were conducted in duplicates. For fed state simulation using biorelevant media FeSSGF and FeSSIF were used instead of SGF and SIF and gastrointestinal digestion of OPE was simulated according to the procedure described above. For simulation of food effects on bioaccessibility of polyphenols using SFM, SFM was mixed with SSF in 1:1 ratio in the beginning of the experiment and gastrointestinal digestion of OPE was simulated according to the procedure described above. Total amount of polyphenols/HTS/TS in OPE was determined by dissolving dry OPE in distilled water. Bioaccessibility of polyphenols was calculated according to Equation (1):

(1) $$\mathrm{Bioaccessibility}\;\left(\%\right)\;=\;\frac{\mathrm{bioaccessible}\;\mathrm{amount}}{\mathrm{total}\;\mathrm{amount}}\;\times \;100$$

Impact of food on bioaccessibility of investigated compounds was expressed as relative bioaccessibility, that compares the bioaccessibility after digestion with food with bioaccessibility from OPE digested without the presence of food using SSF/SGF/SIF, and it was calculated according to Equation (2):

(2) $$\mathrm{Relative}\;\mathrm{bioaccessibility}\;\left(\%\right)\;=\;\frac{\mathrm{bioaccessibility}\;\mathrm{with}\;\mathrm{food}\;\left(\%\right)}{\mathrm{bioacessibility}\;\mathrm{without}\;\mathrm{food}\;\left(\%\right)}\;\times \;100$$

### 2.6. Transepithelial Transport of HTS and TS in Caco-2 Monolayers

For investigation of transepithelial transport human epithelial colorectal adenocarcinoma cell line (Caco-2) was used. Caco-2 cells (ATCC) were cultured in DMEM supplemented with 10% heat-inactivated FBS, 1% NEAA, and 1% A/A. Cell cultures were maintained at 37 °C, in a humidity saturated atmosphere consisted of 5% CO_2_ (Sanyo MCO 20AIC CO_2_ Incubator, Osaka, Japan). Medium was changed every 2 days. Cells were passaged at 80–90% confluence. The study of transepithelial transport was conducted by using 40 µg/mL of TS and HTS diluted in HBSS (MIX) in the presence of one of the following substances: 10 mM glucose (GLU); 0.25% nonessential amino acids (NEAA); 0.125% nonessential amino acids (NEAA2x); 2.4 mg/mL hydroxypropyl β cyclodextrin (HPB); 1% cellulose (alba fiber); 1% inulin (inulin). The highest non-toxic concentration of samples was determined by MTT assay [24]. The 3 × 10^5^ cells/well were seeded in 96-well plates (Thermo Fisher Scientific 130188, Rochester, NY, USA) and grown until reaching confluence (approximately 48 h). The medium was aspirated, and the cells washed with 100 μL PBS/well. Cells were incubated for 4 h with either 100 μL of samples or Hank’s balanced salt solution (HBSS) for positive control or 350 µM tBOOH for negative control. Samples were then removed, and cells were washed with 100 μL PBS/well. Cell viability was assessed by the addition of 40 μL of MTT 0.5 mg/mL (diluted in PBS) and incubation for 3 h at 37 °C, followed by dissolution of the formazan crystals in 170 μL of DMSO. Absorbance (A) was measured at 490 nm and cell viability was expressed as percentage (%) relative to the positive control according to Equation (3). Blank absorbance was measured in wells containing MTT without cells.

(3) $$\%\;\mathrm{viability}\;=\;\frac{\mathrm{Asample}\;–\;\mathrm{Ablank}}{\mathrm{Acontrol}\;–\;\mathrm{Ablank}}\times 100$$

Transepithelial transport was investigated in 12-well plate with Transwell^®^ permeable supports (Costar 3401, Corning Incorporated, Kennebunk, ME, USA) [25]. The 3 × 10^5^ cells were seeded per well and maintained at 37 °C, in a humidity saturated atmosphere consisted of 5% CO_2_. Cells were grown for 21 days in order to form a differentiated monolayer on filters. The medium was warily aspirated (Gilson Safe Aspiration Station, Middleton, WI, USA) and replaced with fresh one every 2 days. The added volume was 0.5 mL in the apical and 1.5 mL in the basolateral compartment. Monolayer integrity was routinely checked by determining transepithelial electrical resistance (TEER) during the cell growth, before and after the transport experiment. Electrical resistance (ER) was measured with STX2 and Epithelial Volt/Ohm Meter (EVOM) (World Precision Instruments Inc., Sarasota, FL, USA). TEER, defined as ER per area, was calculated according to Equation (4).

(4) $$\mathrm{TEER}\;=\;\left(R\;–\;120\;\Omega \right)\;\times \;1.12{\;\mathrm{cm}}^{2}$$

where TEER is transepithelial electrical resistance, 120 Ω is resistance of the cell free-well (blank), 1.12 cm ^2^ is the filter surface.

To evaluate transepithelial permeability, medium was aspirated from both apical and basolateral chambers, and washed twice with pre-warmed PBS. Then, 0.5 mL of the sample or MIX (TS and HTS (40 µg/mL)) was added to the apical chamber and 1.5 mL of the HBSS to the basolateral chamber of each well. Samples were incubated at 100 rpm and 37 °C for 2 h in a shaker (Biosan Incubator ES-20/60, Riga, Latvia). The initial amount of HTS and TS added to a monolayer and the amount in basolateral compartment after the 2 h incubation of Caco-2 cells with the samples was determined by HPLC-FLD. The transepithelial transport of HTS and TS was expressed as % of the amount applied on cell monolayer according to Equation (5).

(5) $$\%=\frac{\mathrm{amount}\;\mathrm{in}\;\mathrm{basolateral}\;\mathrm{compartment}}{\mathrm{initial}\;\mathrm{amount}}\times 100$$

### 2.7. Statistical Analysis

All experiments were run in triplicate unless otherwise stated. Data were statistically tested by one-way analysis of variance (ANOVA), followed by Tukey’s multiple comparisons test. Results were expressed as average value and standard deviation. *p* ≤ 0.05 was considered statistically significant unless otherwise noted. GraphPad^®^ Prism Software (San Diego, CA, USA) was used for statistical analysis.

## 3. Results and Discussion

### 3.1. Impact of Food-Induced Physiological Changes in Gastrointestinal System on Bioaccessibility of OPE Polyphenols

In order to assess the gastrointestinal bioaccessibility of particular compound by an in vitro approach it is essential to mimic the conditions in the gastrointestinal tract as closely as possible. There are several types of in vitro digestion methods that are commonly used for food, but static models, which use a constant ratio of food to enzymes and electrolytes, and a constant pH for each digestive phase, are used most often. However, the experimental conditions in static digestion simulation protocols differ significantly, depending on the purpose of the study and type of analyte/food matrix analyzed. This results with the large number of digestion protocols, which differ considering pH, duration of digestion phases, enzyme concentration and activity, and composition of simulated digestive fluids [26], resulting in questionable relevance of obtained data. Therefore, recently, international consensus was reached in terms of determining optimal conditions that should be applied for in vitro digestion simulation in static models. Such optimized and standardized procedure was applied in this investigation [23].

**Figure 1:**
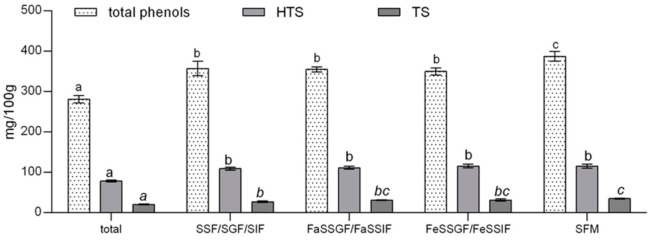
Impact of fed-state on gastrointestinal bioaccessibility of total polyphenols, hydroxytyrosol, and tyrosol from olive pomace extract. SSF—simulated salivary fluid; SGF—simulated gastric fluid; SIF—simulated intestinal fluid; FaSSGF—fasted state simulated gastric fluid; FaSSIF—fasted state simulated intestinal fluid; FeSSGF—fed state simulated gastric fluid; FeSSIF—fed state simulated intestinal fluid; SFM—standardized food matrix; HTS—hydroxytyrosol; TS—tyrosol. Data belonging to the same group are marked with the same letter belong to the same statistical group (*p* ≤ 0.05).

Results presented in Figure 1 show that bioaccessibility of total phenols, HTS and TS is slightly but significantly increased during gastrointestinal digestion, due to liberation of polyphenolic compounds from complex food matrix (281.0 to 357.5 mg/100g; 78.4 to 109.3 mg/100g; and 20.7 to 27.4 mg/100g, respectively). This is consistent with available literature data stating that during the gastric phase of digestion the majority of polyphenols is released from food matrix where they originally formed covalent bonds with carbohydrates of the cell wall, proteins, or one with another [10]. Our previous investigation that focused on the impact of cyclodextrin encapsulation on bioaccessibility of HTS and TS also showed that both, HTS and TS are stable in the gastrointestinal tract, and that their concentrations remain the same or increase during gastrointestinal digestion [8].

As mentioned before, the effect of food on bioaccessibility of particular compounds can be assessed by several methodological approaches, including biorelevant dissolution testing. Food effects on drug absorption are generally better predicted when using biorelevant media containing bile salts and lecithin as compared to the traditional media (SGF and SIF). Namely, the presence of food in gastrointestinal tract changes the conditions in the stomach and intestines, in terms of pH, osmolarity, and composition of digestion fluids, which can significantly influence the pharmacokinetic properties and bioaccessibility of particular compounds [27]. Mentioned differences of the fed/fasted gastric/intestinal state, can be mimicked by fed–fasted state biorelevant media with sufficient accuracy, and although usually used in biorelevant dissolution testing of drugs, they can be applied in the field of nutraceutical formulation and testing. Food effects on bioaccessibility of OPE polyphenols, HTS and TS can be assessed by comparing the fasted state simulated gastric fluid (FaSSGF)/ fasted state simulated intestinal fluid (FaSSIF) and FeSSGF/FaSSIF data presented in Figure 1. Presented data clearly show that changes of pH and digestion fluid composition induced by presence of food in gastrointestinal tract do not influence the bioaccessibility of OPE polyphenols, HTS and TS. Moreover, there were no differences between results obtained by simulating digestion with SSF/SGF/SIF and biorelevant fasted state media. Considering the physicochemical characteristics of hydroxytyrosol and tyrosol, obtained results were partially expected. Namely, significant food effect can be anticipated based on both solubility and permeability of particular compound as described by the Biopharmaceutics Classification System (BCS) [28,29]. Taking into account high water solubility of HTS and TS (17.4 g/L and 25.3 g/L, respectively) and low apparent permeability assessed by predicted logP values (0.85–1.19 and 0.13–0.89, respectively) HTS and TS could be provisionally classified into class 3, according to BCS classification. BCS Class III (high solubility, low permeability) drugs tend to have negative food effects, but they can only be observed by investigating the interactions of food/food compounds with drug absorption and not biorelevant media bioaccessibility testing [30]. As mentioned before, introduction of SFM can be considered as a step forward in in vitro investigation of food effects on bioaccessibility of bioactive compounds because it takes into account wider spectra of possible bioactive compound–food matrix interactions that might affect pharmacokinetic properties of the particular compound [19]. In comparison to other applied methods, simulation of gastrointestinal digestion with SFM significantly increased bioaccessibility of OPE polyphenols in general but had no effect on HTS and TS bioaccessible fractions. This was partially expected, given the high water solubility and previously established gastrointestinal stability of HTS and TS [8]. Namely, positive food effects are usually associated with nonpolar polyphenols because the presence of fat in the food matrix enhances the formation of micelles during the intestinal digestion phase and increases polyphenol content in the soluble (bioaccessible) digest fraction [10]. Conversely to our results, absorption of tyrosol and hydroxytyrosol was positively influenced by the lipid-rich matrix (olive oil), resulting in 25% greater absorption compared to aqueous solution in the rat model [31]. However, the effect could have been achieved, not due to the lipid matrix, but the formation of HTS during gastrointestinal digestion (due to cleavage of more complex antioxidants (such as oleuropein)) and the protection of HTS and TS by other antioxidants present in the olive oil. Positive effects of food matrix on polyphenol bioavailability can also be attributed to the presence of fermentable fiber (such as pectin) that increase colonic bioavailability of particular polyphenols due to their prebiotic properties. These effects could not be monitored in our study. Therefore, observed slight, but statistically significant positive effects of SFM on phenolic bioaccessibility can likely be attributed to the impact of fat on micellization of nonpolar polyphenols present in OPE.

### 3.2. Gastrointestinal Interactions of HTS and TS with Particular Food

**Figure 2:**
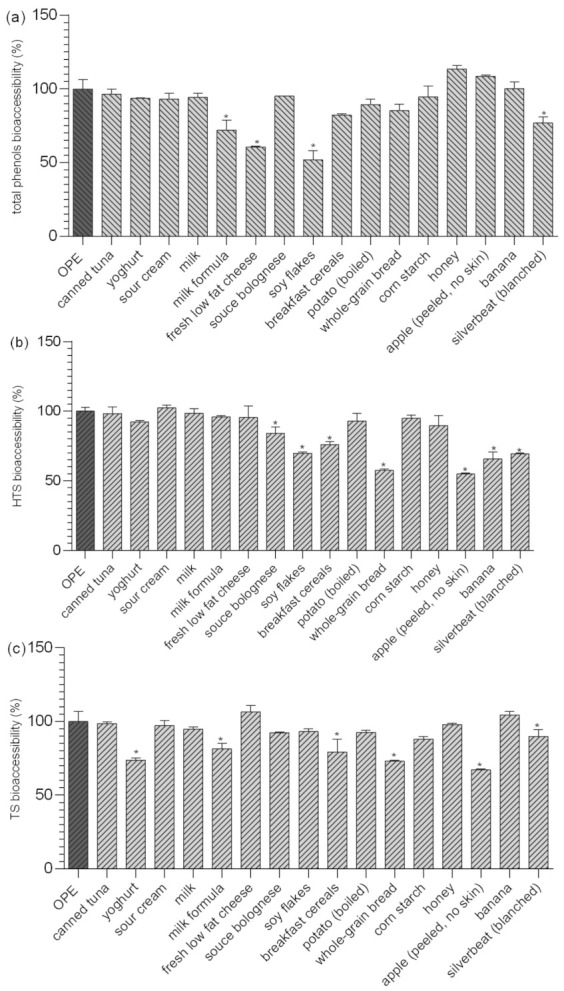
Impact of different foods on relative bioaccessibility* of total polyphenols (**a**), HTS) (**b**) and TS (**c**) from OPE. * Relative bioaccessibility was calculated in relation to in vitro bioaccessibility of total phenols, HTS, and TS from OPE obtained after simulation of gastrointestinal digestion using FaSSIF/FeSSIF. Columns marked with * differ significantly from OPE (*p* ≤ 0.05). OPE—olive pomace extract; FaSSGF—fasted state simulated gastric fluid; FaSSIF—fasted state simulated intestinal fluid; HTS—hydroxytyrosol; TS—tyrosol.

Bioavailability of olive polyphenols from food matrices other than olive oil has rarely been studied. Radić and co-workers [8] investigated the influence of olive pomace matrix and cyclodextrin encapsulation on bioavailability of olive polyphenols, and showed high bioaccessibility, but relatively low permeability, of hydroxytyrosol and tyrosol, which were both negatively affected by olive pomace matrix. Their results confirmed the previous findings of Malapert and co-workers [32], who showed that bioavailability of hydroxytyrosol was higher when it was applied as a pure compound than from Alperujo powder. They also showed that presence of foods (investigated by co-digestion with the test meal) significantly decreased hydroxytyrosol bioaccessibility and intestinal permeability (−20% and −10%, respectively). They supposed that negative food effects arise from the fact that polyphenols form complex bonds with particular food components, and that the investigation of the influence of the food matrix on the bioaccessibility of dietary plant phenols, such as HT, requires including real meal components into in vitro digestion studies [32]. This is consistent with our results (Figure 1), where the comparison of results obtained in fed/fasted biorelevant media showed that changes in pH, ionic strength, or the amount of bile during gastrointestinal digestion are not determining factors for HTS and TS bioaccessibility. However, data presented in Table S5 and Figure 2 clearly show that both bioaccessibility and relative bioaccessibility of HTS and TS can be significantly changed during co-digestion with different foods, and that the extent of observed changes depends on chemical/nutritive composition of food matrices (Table S4). Depending on the food matrix investigated, bioaccessibility of OPE polyphenols was either not affected or it was significantly reduced. The most significant impact was observed after co-digestion of OPE with soy flakes, fresh low-fat cheese, and milk formula (relative bioaccessibility of TP was 51.8%, 60.6%, and 71.9% respectively), foods with relatively high protein content (52%, 12.4%, 10.5%) Bioaccessibility of HTS and TS was also significantly reduced by some of the investigated high-protein foods: soy flakes, sauce Bolognese, breakfast cereals, and whole-grain bread, but the effect was less pronounced than in the case of TP. Observed effects were, at least partially influenced, by polyphenol–protein interactions, well described in scientific literature and reviewed recently in the work of Jakobek [14]. Polyphenols (especially polyhydroxy polyphenols) form non-covalent hydrophobic interactions with proteins, which may subsequently be stabilized by hydrogen bonding [33], and can, among other effects, change the bioavailability of polyphenols. Different authors observed negative effects of milk proteins on bioavailability of polyphenols of different origin [34,35,36,37,38]. The majority of observed negative interactions in literature were observed for casein, which is consistent with our results showing the stronger negative effects of foods with casein content in comparison to other high-protein foods (although total protein content was relatively low). To our knowledge, impact of proteins on bioaccessibility of hydroxytyrosol and tyrosol has not been previously investigated, but it has been shown that hydroxytyrosol can form adducts with food proteins [39]. Obtained results suggest the negative interactions between proteins and polyphenols, but additional effects of matrix components, such as lipids or carbohydrates, must also be considered. For example, despite high protein content, canned tuna or milk formula did not negatively influence bioaccessibility of hydroxytyrosol, maybe partially due to high fat content (15.5 and 27.4%, respectively). Namely, although investigations suggest that interactions between lipids and polyphenols have only a small influence on the polyphenol accessibility for absorption [14], some studies show that when lipids interact with polyphenols, they can “capture” polyphenols and protect them from degradation or forming insoluble complexes in their passage through the gastrointestinal tract [40], or increase their solubility and activity in gastrointestinal tract by forming micelles and promoting emulsification [41]. In our study, such effects were not particularly pronounced, since OPE polyphenols have been proven stable during gastrointestinal digestion and are generally soluble in water [8].

Interactions between polyphenols and available carbohydrates have been described in scientific literature because polyphenols can reduce starch digestion, possibly via amylase inhibition, and inhibit glucose uptake transporters [42]. Far fewer studies have examined the impact of carbohydrates on polyphenol bioavailability and were mostly focused on polyphenol uptake. In our investigation, corn starch and honey (100% and 80% of available carbohydrates) were selected as model foods for investigation of available carbohydrate impact on olive polyphenols bioaccessibility. Relative bioaccessibility of TF, HTS, and TS after co-digestion with corn starch/honey was 94.5/113.6%; 94.9/89.5%, and 88.7/97.8%, and observed differences were not statistically significant (Figure 2).

**Figure 3:**
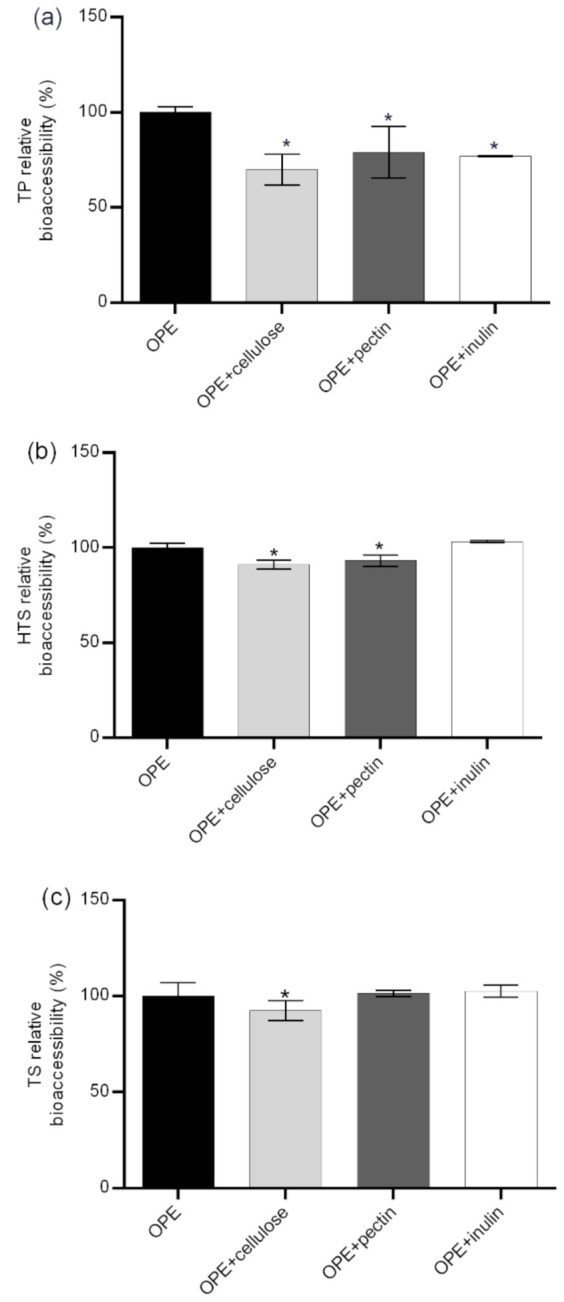
Impact of cellulose, pectin and inulin on relative bioaccessibility* of TP, HTS, and TS from OPE. * Relative bioaccessibility was calculated in relation to in vitro bioaccessibility of TP, HTS, and TS from OPE obtained after simulation of gastrointestinal digestion using FaSSIF/FeSSIF. Columns marked with * differ significantly from OPE (*p* ≤ 0.05). OPE—olive pomace extract; FaSSGF—fasted state simulated gastric fluid; FaSSIF—fasted state simulated intestinal fluid; TP—total phenols; HTS—hydroxytyrosol; TS—tyrosol.

While the negative impacts of indigestible carbohydrates (dietary fiber) on digestibility of fat and bioaccessibility of some minerals and trace elements is well known, their influence on the bioaccessibility of polyphenolic compounds has not been thoroughly investigated. The main direct mechanisms of negative impacts of dietary fiber on polyphenol intestinal availability are an incomplete release of polyphenols from fiber rich fruit and vegetable matrices (i); presence of polyphenols bound to polysaccharides requiring enzymatic hydrolysis to be absorbed (ii); and entrapment of polyphenols by dietary fiber during digestion in the upper intestine (iii) [43]. The possibility of the formation of insoluble polyphenol–dietary fiber complexes during gastrointestinal digestion is particularly important for this investigation since it focused on the bioaccessibility of polyphenols previously isolated from its natural complex matrix. As shown in Figure 2, bioaccessibility of TP was not significantly reduced in the presence dietary fiber containing food (Table S4) except for soy flakes that contained high content of both proteins and fiber (52% and 16%, respectively). On the other hand, a negative impact of fiber was more noticeable in the case of TS and HTS. Bioaccessibility of TS was significantly reduced in the presence of breakfast cereals, wholegrain bread, apple and silver beat, ranging from 67% to 89% in relation to bioaccessibility of OPE consumed with no food. The negative effect of dietary fiber was even more pronounced in the case of HTS. Its relative bioaccessibility was significantly reduced in the presence of all fiber containing foods and ranged from 54.8% up to 84.2%. Pronounced negative effects on hydroxytyrosol bioaccessibility might be explained by the additional hydroxyl group in HTS structure since dietary fiber–polyphenol interactions were observed to appear through non-covalent bonds as electrostatic forces and hydrogen bonds as Van der Waals forces between hydroxyl groups of phenolic compounds and various components of dietary fibers [44]. The negative impact on HTS/TS bioaccessibility was not proportional to the content of dietary fiber in the food matrix probably because it is the consequence of complex interactions of all matrix components and also the chemical composition of the fiber. In order to investigate the gastrointestinal polyphenol–dietary fiber interactions more thoroughly, we additionally simulated gastrointestinal digestion of OPE in the presence of cellulose, pectin and inulin and assessed the impact on the relative bioaccessibility of OPE antioxidants (Figure 3). Presented data show that the impact of dietary fiber on polyphenol bioaccessibility depends on both types of dietary fiber and chemical characteristics of particular polyphenols. All investigated fiber significantly reduced relative bioaccessibility of TP from OPE (69.8–79%), and cellulose showed the strongest negative impact. Relative bioaccessibility of HTS was reduced to 88.1% by cellulose and to 93.2% by pectin and was not influenced by the presence of inulin in the reaction mixture. TS relative bioaccessibility was reduced to 87.4% by cellulose and was not affected by the presence of other dietary fiber. The comparison of fiber-in-food-matrix–polyphenol interactions to pure fiber–polyphenol interactions (Figure 2 and Figure 3, respectively) indicate the significant effect of the food matrix on the type and extent of polyphenol–dietary fiber interactions. Therefore, data on the effect of fiber-rich food on polyphenol bioaccessibility cannot be extrapolated from the data obtained by in vitro digestion of polyphenols with pure dietary fiber. Therefore, in order to guide the design of functional foods enriched with phenolic substances, we should significantly increase the number of in vitro and in vivo studies on the bioavailability of particular phenolic substances in contact with particular foods. This is consistent with the conclusions of Pinarli and co-workers [45], stating that, due to complexity of interactions within the food matrix, every food material and component should be evaluated individually with respect to its interacting phenolic compound. Although HTS/TS-dietary fiber interactions have hardly been investigated, our observations are consistent with the recent findings of Bermúdez-Oria and co-authors [46], who confirmed the formation of predominant hydrogen–bonding interactions between HTS and strawberry dietary fiber. Tomas and co-authors [47] noticed modulation of polyphenols profile of blackberry purees by soluble dietary fiber (inulin or pectin), during a simulated in vitro gastrointestinal digestion and large intestine fermentation process, and explained it by the interactions of dietary fiber and polyphenols.

### 3.3. Impact of Food on Intestinal Permeability of TP, HTS, and TS

**Figure 4:**
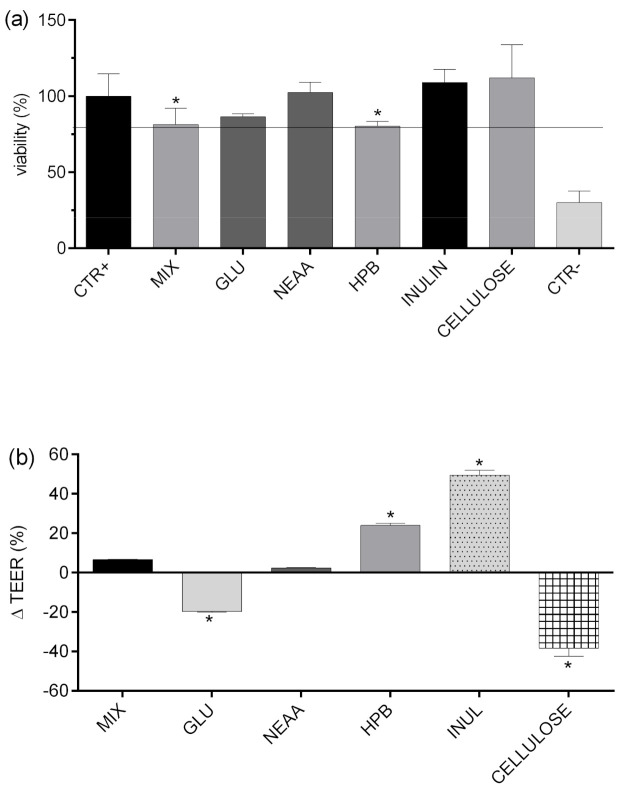
Viability (%) of Caco-2 cell monolayer determined by MTT test after exposure to analyzed food fractions (**a**). Relative change (%) of TEER values during the experiment (**b**). positive control (CTR+)—HBSS; MIX—TS + HTS (40 mg/L); EDTA (10μM); GLU—glucose (10 mM); NEAA—non-essential amino acids (0.25%); HPB—hydroxypropyl beta-cyclodextrin (2.4 g/L); CELLULOSE—cellulose (1%); INULIN—inulin (1%); CTR^—^tBOOH (350 µM); TEER—transepithelial electrical resistance. Columns marked with * differ significantly from the reference column: (CTR+ (**a**); MIX (**b**)).

The influence of MIX and investigated food components on the viability of human epithelial colorectal adenocarcinoma cells Caco-2 was determined by using the MTT assay (Figure 4a) and by measuring TEER values, before and after the experiment (Figure 4b), as previously described. The results presented in Figure 4 show that cell viability was significantly decreased when cells were treated with the MIX and HPB, which is consistent with previously obtained data [48]. Although significantly decreased in relation to positive control (CTR+), viability of monolayers treated with MIX and HPB was above 80% (81.4 and 80.3%, respectively) and, therefore, suitable for further analysis. Moreover, it is evident from Figure 4b that, even though tested food matrices do not produce significant cytotoxic effects, they can significantly affect the intestinal barrier, and may increase bilayer permeability or promote barrier integrity. Those effects are achieved through different mechanisms: affecting mucus permeation, paracellular permeation, bilayer permeability, or active transport and efflux transport [12,49]. As shown previously, food matrix can significantly affect intestinal permeability of polyphenols through formation of soluble or insoluble complexes, resulting with positive or negative effects on bioaccessibility. Therefore, the actual intestinal permeability of a particular compound is the result of all of the above-mentioned interactions and, because of that, it is very hard to predict.

At the moment, available data on the impact of food matrix/nutrients on intestinal permeability of polyphenols are scarce. Recently, Nogueira Mendes and co-authors [50] showed that intestinal permeability of guarana catechins and procyanidins was not significantly affected by macronutrients (casein, starch and oil). On the other hand, transepithelial transport of indicaxanthin and betanin was negatively affected by particular food matrix components while intestinal transport of green tea catechins was positively affected by milk proteins, probably due to impact of milk on tight junction permeability [51,52]. The mentioned results point out the significance of chemical characteristics of targeted molecules and the exact composition of the food matrix in predicting food matrix effects, as well as the necessity of focused and tailored research.

**Figure 5:**
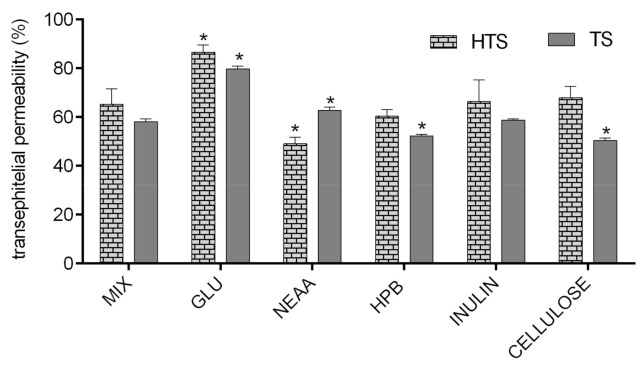
Permeability (%) of HTS and TS in Caco-2 cell monolayer (the amount of TS/HTS in basolateral compartment was expressed as the percentage of the amount applied to Caco-2 cell monolayer). MIX-TS + HTS (40 mg/L); GLU—glucose (10 mM); NEAA—non-essential amino acids (0.25%); HPB—hydroxypropyl beta-cyclodextrin (2.4 g/L); APPLE FIBRE—apple fiber (1%); INULIN (1%). Columns belonging to the same series marked with * differ significantly (p ≤ 0.05) from the reference column (MIX).

In order to investigate the impact of glucose, amino acids, dietary fiber (inulin and apple fiber), and HPB (excipient often used in nutraceutical production) on transepithelial permeability of HTS and TS, they were applied to Caco-2 monolayers in combination, and their permeability was compared to permeability of HTS and TS from pure mixture (MIX). As presented in Figure 5, permeability of HTS and TS was mostly unaffected by tested compounds, with few exceptions. First, glucose significantly improved permeability of both HTS and TS. The mechanisms of such interaction are unknown and beyond the scope of this investigation, but certain assumptions can be made based on available literature data. DeSouza and co-authors [53] investigated Caco-2 cell permeability was evaluated in isotonic media containing high (25 mM) or physiological (5.5 mM) glucose concentrations, and concluded that high extracellular glucose concentration in isotonic media significantly alters physical barrier properties of Caco-2 cell monolayers, which predominantly affects transepithelial transport of solutes permeating the cell barrier by paracellular and transcellular passive diffusion through decreasing TEER of cell monolayers, increasing membrane fluidity. Their results are in consistence with data presented in Figure 4B showing that glucose significantly decreased TERR of Caco-2cell monolayers (20%).

The TEER of cell monolayers was also negatively affected by cellulose (40%, but it did not result in increased permeability of HTS/TS. This is probably due to formation of insoluble complexes with HTS/TS, as shown in Figure 3, where cellulose decreased bioaccessibility of both investigated phenolic compounds. These observations are consistent with data obtained by Bermudez-Oria and co-authors [46], who showed that olive polyphenols form insoluble complexes with dietary fibers. On the other hand, inulin had the opposite effects on HTS and TS intestinal permeability. As shown in Figure 4B, it significantly increased TEER (40%). This observation is consistent with available literature data showing that fructooligosaccharides can improve intestinal barrier function through short fatty acid formation [54,55], but such effects could not have been noticed in our investigation (that did not include the step of microbial fermentation of fermentable fiber). However, our data show that inulin can have additional, direct impacts on Caco-2 cell permeability, and it resulted in a significant reduction of HTS transepithelial permeability.

## 4. Conclusions

Changes in pH, ionic strength, enzyme activity, or the amount of bile due to presence of food in digestive system, are not determining factors for HTS and TS bioaccessibility, but can slightly improve bioaccessibility of other OPE polyphenols. Soy, milk formula, cheese, and breakfast cereals exerted negative effects on HTS and TS bioaccessibility, probably due to negative HTS/TS-casein and HTS/TS-dietary fiber interactions. Particular nutrients significantly influenced the permeability of Caco-2 cell monolayer (without exerting cytotoxic effects); it was increased by glucose and cellulose while inulin improved monolayer barrier function. Those effects significantly affected transepithelial transport of HTS and TS. Our results provide new insights into factors affecting bioaccessibility and transepithelial permeability of HTS and TS and point out the complexity of polyphenol interactions within the food matrix. Therefore, in the process of formulating functional foods enriched with phenolic substances, every food matrix should be evaluated individually, with respect to its interacting phenolic compounds.

## Supplementary Materials

The following are available online at https://www.mdpi.com/2304-8158/9/12/1831/s1. Table S1. Composition of simulated salivary fluid (SSF), simulated gastric fluid (SGF) and simulated intestinal fluid (SIF); Table S2. Composition of standardized food model (SFM); Table S3. Chemical composition of OPE; Table S4. Macronutritive composition of food matrices used for investigations of gastrointestinal interactions with hydroxytyrosol and tyrosol; Table S5. Impact of different foods and dietary fibre on relative bioaccessibility* of HTS, TS and total polyphenols from OPE.
