# Supplementary material for: Food (Matrix) Effects on Bioaccessibility and Intestinal Permeability of Major Olive Antioxidants
Source: Foods. 2020 Dec 9;9(12):1831. doi: 10.3390/foods9121831 (PMC7764665; doi:10.3390/foods9121831)
Supplement: Supplementary file 1 [file foods-09-01831-s001.zip › Table S1.docx]

|  | Volume of stock solution needed for preparation of 200 mL of SSF/SGF/SIF | | |
| --- | --- | --- | --- |
|  | **SSF** | **SGF** | **SIF** |
|  | pH 7 | pH 3 | pH 7 |
| Stock solution | V/mL | V/mL | V/mL |
| KCl | 7.55 | 3.455 | 3.4 |
| KH_2_PO_4_ | 1.85 | 0.45 | 0.4 |
| NaHCO_3_ | 3.4 | 6.25 | 21.25 |
| NaCl | - | 5.9 | 4.8 |
| MgCl_2_(H_2_O)_6_ | 0.25 | 0.2 | 0.55 |
| (NH_4_)_2_CO_3_ | 003 | 0.25 | - |
| Adjustment of pH | V/mL | V/mL | V/mL |
| 1 M NaOH | - | - | - |
| 1 M HCl | 0.045 | 0.65 | 0.35 |

**Table S1.** Composition of simulated salivary fluid (SSF), simulated gastric fluid (SGF) and simulated intestinal fluid (SIF)
