# Supplementary material for: Food (Matrix) Effects on Bioaccessibility and Intestinal Permeability of Major Olive Antioxidants
Source: Foods. 2020 Dec 9;9(12):1831. doi: 10.3390/foods9121831 (PMC7764665; doi:10.3390/foods9121831)
Supplement: Supplementary file 1 [file foods-09-01831-s001.zip › Table S2.docx]

**Table S2.** Composition of standardized food model (SFM)

| Macronutrient | Ingredient | g/100g of SFM |
| --- | --- | --- |
| Protein | sodium caseinate | 3.44 |
| Available carbohydrate | sucrose | 4.57 |
|  | starch | 5.15 |
| Dietary fibre | apple pectin | 0.7 |
| Fat | sunflower oil | 3.42 |
| Minerals | sodium chloride | 0.53 |
