# Supplementary material for: Food (Matrix) Effects on Bioaccessibility and Intestinal Permeability of Major Olive Antioxidants
Source: Foods. 2020 Dec 9;9(12):1831. doi: 10.3390/foods9121831 (PMC7764665; doi:10.3390/foods9121831)
Supplement: Supplementary file 1 [file foods-09-01831-s001.zip › Table S3.docx]

| **Chemical analysis** | **g/100g OPE** |
| --- | --- |
| **Moisture^*^** | 4.1 |
| **Ash^*^** | 10.2 |
| **Protein^*^** | 22.7 |
| **Fat^*^** | 0.9 |
| **Total carbohydrates^**^** | 55.6 |
| **Total polyphenols^***^** | 6.5 |

**Table S3.** Chemical composition of OPE

*^*^determined by standard AOAC methods; ^**^determined by difference;^***^determine by Folin-Ciocalteu method and expressed as gallic acid equivalent*
