# Supplementary material for: Food (Matrix) Effects on Bioaccessibility and Intestinal Permeability of Major Olive Antioxidants
Source: Foods. 2020 Dec 9;9(12):1831. doi: 10.3390/foods9121831 (PMC7764665; doi:10.3390/foods9121831)
Supplement: Supplementary file 1 [file foods-09-01831-s001.zip › Table S4.docx]

| Table S4. Macronutritive composition of food matrices used for investigations of gastrointestinal interactions with hydroxytyrosol and tyrosol | | | | | | | |
| --- | --- | --- | --- | --- | --- | --- | --- |
|  | **protein** | **fat** | **available carbohydrates** | | **total fibre** | **soluble fibre** | **insoluble fibre** |
|  | g/100g of eddible part | | | | | | |
| canned tuna^*^ | 21.5 | 15.5 | | 0.0 | 0.0 | 0.0 | 0.0 |
| yoghurt^*^ | 4.3 | 2.8 | | 5.1 | 0.0 | 0.0 | 0.0 |
| sour cream^*^ | 3.0 | 12.0 | | 4.4 | 0.0 | 0.0 | 0.0 |
| milk^*^ | 3.3 | 3.2 | | 4.6 | 0.0 | 0.0 | 0.0 |
| milk formula^*^ | 10.5 | 27.4 | | 62.1 | 0.0 | 0.0 | 0.0 |
| fresh low fat cheese^*^ | 12.4 | 1.0 | | 2.7 | 0.0 | 0.0 | 0.0 |
| souce Bolognese^*^ | 6.2 | 5.3 | | 7.1 | 1.8 | 1.2 | 0.4 |
| soy flakes^*^ | 52.0 | 6.0 | | 4.0 | 16.0 | 0.0 | 16.0 |
| breakfast cereals^*^ | 12.8 | 2.3 | | 68.9 | 11.2 | 1.1 | 10.1 |
| potato (boiled)^**^ | 0.9 | 0.2 | | 17.6 | 2.8 | 1.6 | 1.2 |
| whole grain bread^*^ | 8.0 | 2.0 | | 46.0 | 2.7 | 2.5 | 0.2 |
| corn starch | 0.0 | 0.0 | | 100.0 | 0.0 | 0.0 | 0.0 |
| honey^**^ | 0.4 | 0.0 | | 80.0 | 0.0 | 0.0 | 0.0 |
| apple (peeled, no skin)^**^ | 0.3 | 0.1 | | 12.6 | 1.4 | 1.1 | 0.3 |
| banana^**^ | 1.1 | 0.3 | | 19.2 | 2.8 | 0.7 | 2.1 |
| silverbeat (blanched)^**^ | 1.3 | 0.1 | | 1.5 | 1.2 | 0.3 | 0.9 |
| *cellulose fiber* | - | - | | - | 100 | - | 100 |
| *pectin* | - | - | | - | 100 | 100 | - |
| *inulin* | - | - | | - | 100 | 100 | - |

*^*^nutrition data tables of the product provided by the manufacturer/supplier; ^**^data taken from nutrition data tables (Kaić Rak i Antonić, 1990)*
