# Supplementary material for: Food (Matrix) Effects on Bioaccessibility and Intestinal Permeability of Major Olive Antioxidants
Source: Foods. 2020 Dec 9;9(12):1831. doi: 10.3390/foods9121831 (PMC7764665; doi:10.3390/foods9121831)
Supplement: Supplementary file 1 [file foods-09-01831-s001.zip › Table S5.docx]

|  | **HTS** | **TS** | **total phenols** |
| --- | --- | --- | --- |
|  | realtive bioaccessibility (%) | | |
| **OPE** | 100.0 ± 2.86 | 99.9 ± 6.75 | 99.9 ±6.34 |
| **canned tuna** | 98.3 ± 4.92 | 98.5 ± 1.12 | 96.4 ± 3.48 |
| **yoghurt** | 92.5 ± 0.82 | 73.8 ± 1.42 | 93.7 ± 0.36 |
| **sour cream** | 102.3 ± 2.18 | 97.4 ± 3.34 | 93.1 ± 3.89 |
| **milk** | 98.6 ± 3.11 | 94.6 ± 1.63 | 94.1 ± 2.76 |
| **milk formula** | 95.9 ± 0.96 | 81.3 ± 3.95 | 71.9 ± 7.01 |
| **fresh low fat cheese** | 95.5 ± 8.16 | 106.4 ± 4.35 | 60.6 ± 0.63 |
| **souce bolognese** | 84.2 ± 4.61 | 92.4 ± 0.46 | 95.1 ± 0.14 |
| **soy flakes** | 69.8 ± 1.06 | 93.0 ± 1.93 | 51.8 ± 6.36 |
| **breakfast cereals** | 76.0 ± 2.30 | 79.3 ± 8.70 | 82.2 ± 0.95 |
| **potato (boiled)** | 92.8 ± 5.88 | 92.4 ± 1.64 | 89.5 ± 3.44 |
| **whole-grain bread** | 57.4 ± 0.97 | 73.0 ± 0.66 | 85.2 ± 4.40 |
| **corn starch** | 94.9 ± 2.19 | 88.1 ± 1.66 | 94.5 ± 7.45 |
| **honey** | 89.5 ± 7.26 | 97.8 ± 0.91 | 113.6 ± 2.30 |
| **apple (peeled, no skin)** | 54.8 ± 0.72 | 67.2 ± 0.55 | 108.6 ± 0.87 |
| **banana** | 65.8 ± 5.05 | 104.5 ± 2.15 | 100.2 ± 4.48 |
| **silverbeat (blanched)** | 69.3 ± 0.79 | 89.9 ± 4.59 | 76.8 ± 3.99 |
| **cellulose** | 108.2 ± 2.24 | 107.4 ± 7.05 | 69.8 ± 8.06 |
| **pectin** | 93.2 ± 3.04 | 101.5 ± 1.64 | 79.0 ± 13.56 |
| **inulin** | 103.3 ± 0.6 | 102.6 ± 3.17 | 76.8 ± 0.24 |

**Table S5.** Impact of different foods and dietary fibre on relative bioaccessibility^*^ of HTS, TS and total polyphenols from OPE.

*^*^relative bioaccessibility was calculated in relation to in vitro bioacessibility of total phenols, HTS and TS from OPE obtained after simulation of gastrointestinal digestion using FaSSIF/FeSSIF. OPE-olive pomace extract; HTS-hydroxytyrosol; TS-tyrosol.*
